# Impact of circulating tumor DNA mutant allele fraction on prognosis in *RAS*‐mutant metastatic colorectal cancer

**DOI:** 10.1002/1878-0261.12547

**Published:** 2019-07-31

**Authors:** Elena Elez, Chiara Chianese, Enrique Sanz‐García, Erica Martinelli, Alba Noguerido, Francesco Mattia Mancuso, Ginevra Caratù, Judit Matito, Julieta Grasselli, Claudia Cardone, Riziero Esposito Abate, Giulia Martini, Cristina Santos, Teresa Macarulla, Guillem Argilés, Jaume Capdevila, Ariadna Garcia, Nuria Mulet, Evaristo Maiello, Nicola Normanno, Frederick Jones, Josep Tabernero, Fortunato Ciardello, Ramon Salazar, Ana Vivancos

**Affiliations:** ^1^ Department of Medical Oncology Vall d'Hebron Institute of Oncology Barcelona Spain; ^2^ Department of Medical Oncology Vall d' Hebron University Hospital, Universitat Autònoma de Barcelona Spain; ^3^ Cancer Genomics Group Vall d'Hebron Institute of Oncology Barcelona Spain; ^4^ Medical Oncology, Department of Clinical and Experimental Medicine ‘F. Magrassi' Università della Campania ‘L. Vanvitelli' Napoli Italy; ^5^ Department of Medical Oncology, Catalan Institute of Oncology Universitat de Barcelona, L'Hospitalet Spain; ^6^ Cell Biology and Biotherapy Unit Istituto Nazionale Tumori ‘Fondazione Giovanni Pascale' IRCCS Napoli Italy; ^7^ Medical Oncology IRCCS Casa Sollievo della Sofferenza San Giovanni Rotondo (Foggia) Italy; ^8^ Sysmex Inostics Mundelein IL USA

**Keywords:** circulating tumor DNA, MAF, metastatic colorectal cancer, prognostic biomarker, RAS analysis

## Abstract

Despite major advances in the treatment of metastatic colorectal cancer (mCRC), the survival rate remains very poor. This study aims at exploring the prognostic value of *RAS*‐mutant allele fraction (MAF) in plasma in mCRC. Forty‐seven plasma samples from 37 *RAS*‐mutated patients with nonresectable metastases were tested for *RAS* in circulating tumor DNA using BEAMing before first‐ and/or second‐line treatment. *RAS* MAF was correlated with several clinical parameters (number of metastatic sites, hepatic volume, carcinoembryonic antigen, CA19‐9 levels, primary site location, and treatment line) and clinical outcome [progression‐free survival (PFS) and overall survival (OS)]. An independent cohort of 32 patients from the CAPRI‐GOIM trial was assessed for clinical outcome based on plasma baseline MAF. *RAS* MAF analysis at baseline revealed a significant correlation with longer OS [Hazard ratios (HR) = 3.514; *P* = 0.00066]. Patients with lower MAF also showed a tendency to longer PFS, although not statistically significant. Multivariate analysis showed *RAS* MAFs as an independent prognostic factor in both OS (HR = 2.73; *P* = 0.006) and first‐line PFS (HR = 3.74; *P* = 0.049). Tumor response to treatment in patients with higher MAF was progression disease (*P = *0.007). Patients with low MAFs at baseline in the CAPRI‐GOIM group also showed better OS [HR = 3.84; 95% confidence intervals (CI) 1.5–9.6; *P* = 0.004] and better PFS (HR = 2.5; 95% CI: 1.07–5.62; *P* = 0.033). This minimally invasive test may help in adding an independent factor to better estimate outcomes before initiating treatment. Further prospective studies using MAF as a stratification factor could further validate its utility in clinical practice.

**Abbreviations**
CEA: carcinoembryonic antigen
cfDNA: circulating free DNA
ctDNA: circulating tumor DNA
HR: hazard ratio
MAF: mutant allele fraction
mCRC: metastatic colorectal cancer
OS: overall survival
PFS: progression‐free survival

## 1 Introduction

The past two decades have witnessed significant progress in the treatment of metastatic colorectal cancer (mCRC) partly due to a better selection of therapy based on the tumor RAS mutation status. Nonetheless, the 5‐year survival rate in mCRC patients remains poor (Siegel *et al.*, 2017). The large variability in survival shows that current routine prognostic evaluation of mCRC is insufficient and needs to be improved, for both resectable and nonresectable metastases. The development of reliable prognostic biomarkers is an increasingly pertinent tool in this setting.

In mCRC, the detection of circulating tumor DNA (ctDNA) is an emerging alternative to detect mutations, thus avoiding biopsies from primary or metastatic sites. We and others previously reported ~ 90% concordance of *RAS*‐mutant status in paired plasma and tissue samples, as well as its predictive value in plasma for anti‐EGFR therapy response (Bettegowda *et al.*, 2014; Grasselli *et al.*, 2017; Siravegna *et al.*, 2015). Mutant allele fractions (MAFs) are a measure of the percentage of mutant alleles within the totality of alleles in any given sample. MAF estimations of driver genes have shown important clinical implications in various settings. In a retrospective analysis of the CRYSTAL trial (Van Cutsem *et al.*, 2011), mCRC patients with tumor *RAS* MAFs between 0.1% and < 5% were more likely to benefit from the addition of cetuximab to FOLFIRI. Likewise, resistance to anti‐EGFR therapies in mCRC with *KRAS* MAFs < 1% (Azuara *et al.*, 2016; Laurent‐Puig *et al.*, 2015) and longer benefit with tyrosine kinase inhibitor therapy were associated with higher MAFs in *EGFR*‐mutated lung cancer patients (Ono *et al.*, 2014; Zhou *et al.*, 2011).

The potential prognostic value of plasma MAFs in mCRC has not been well established yet. Interestingly, we and others have observed that *RAS* MAF showed a trend to lower overall survival (OS) when plasma levels were above a cutoff of 10% and 1%, respectively, although the population was heterogeneous in terms of treatment and time of analysis (El Messaoudi *et al.*, 2016; Siravegna *et al.*, 2017; Vidal *et al.*, 2017). Of note, plasma was obtained at different disease stages and timing on treatment.

To better define the predictive nature of *RAS* MAF levels, we performed a study in a homogeneous group of patients with plasma samples collected systematically prior to the first or second treatment line, to correlate *RAS‐*mutant MAFs with clinical parameters and to determine the impact of *RAS*‐mutant MAF on OS and progression‐free survival (PFS) in these disease settings. We also included an independent cohort from the CAPRI‐GOIM trial that was assessed for clinical outcome based on plasma baseline MAF (Ciardiello *et al.*, 2014; Normanno *et al.*, 2017).

## 2 Materials and methods

### 2.1 Study design

This multicentric study included both retrospective and prospective patients: Retrospective patients were recruited from two Spanish hospitals (Vall d'Hebron University Hospital and Catalan Institute of Oncology, Duran I Reynals); prospective patients were recruited from the Vall d'Hebron University Hospital only. Additionally, an independent cohort of first‐line patients derived from the CAPRI‐GOIM trial (registration number: 2009‐014041‐81) were also included. The study was approved by the ethics committee of all hospitals, and all patients signed written informed consent. This study was conducted in accordance with the Declaration of Helsinki and Good Clinical Practice guidelines.

### 2.2 Patient characteristics

Patients from the TTD ULTRA clinical trial (NCT01704703) were included. Of 110 mCRC plasma samples screened, 62 (56%) were identified as *RAS‐*mutated by BEAMing in plasma. To obtain a homogeneous study population, we excluded patients with liver‐limited resected metastases, leaving 41 plasma samples (37%) from 37 *RAS*‐mutated patients with nonresectable metastases for analysis (Fig. S1); of them, 29 samples were prior to first‐line therapy and 12 prior to second‐line treatment (Table S1). Baseline characteristics, number and location of metastasis, and number and description of previous lines of therapy are summarized in Table 1.

**Table 1:** Patient characteristics.

	First‐line (*N = *29; (%)	Second‐line (*N = *12; %)	CAPRI‐GOIM (*N* = 33; %)
Gender			
Male	19 (65)	7 (58)	16 (48)
Female	20 (35)	5 (42)	17 (52)
*RAS‐mutated*			
KRAS 12	16 (55)	7 (58)	19 (58)
KRAS 13	6 (21)	3 (25)	2 (6)
KRAS (others)	3 (10)	1 (8)	7 (21)
NRAS 12	1 (4)	0	2 (6)
NRAS 13	0	0	0
NRAS (others)	3 (10)	1 (8)	3 (9)
M1 metastatic sites			
1	10 (35)	1 (8)	17 (52)
2	16 (55)	7 (58)	14 (42)
3+	3 (10)	4 (33)	2 (6)
Primary site			
Right	9 (31)	5 (42)	7 (21)
Left	12 (41)	0	16 (48)
Rectum	8 (28)	7 (58)	10 (31)
Treatment			
FOLFOX	26 (89)	3 (25)	
FOLFIRI	1 (4)	7 (58)	
Antiangiogenics	9 (31)	5 (42)	
Others^a^	3 (10)	2 (17)	

a First‐line: 5‐fluorouracil (5‐FU) and capecitabine; Second‐line: irinotecan.

The CAPRI‐GOIM trial, a nonprofit academic, open‐label, multicenter study, enrolled 340 mCRC patients, *KRAS* exon‐2 wild‐type, according to local pathology assessment, treated with FOLFOX plus cetuximab vs FOLFOX at progression to first‐line FOLFIRI plus cetuximab (Eudract number: 2009‐014041‐81) (Normanno *et al.*, 2017). Of these, 33 patients were found mutated according to their plasma sample and thus used in this study as an independent validation set (Table S1) (Normanno *et al.*, 2017). One patient was excluded for analysis due to lack of follow‐up data.

### 2.3 Sample collection

Blood samples (4 mL) were collected in CellSave® Preservative Tubes (Menarini‐Silicon Biosystems, Bologna, Italy), and plasma was isolated within 48 h. For nontrial patients, 10 mL of blood was collected in EDTA tubes and plasma was isolated within 1 h. A two‐step centrifugation was performed with blood initially centrifuged for 10 min at 1600 ***g*** at room temperature. Supernatant was collected, avoiding the buffy coat, and then centrifuged again for 10 min at room temperature at 3000 ***g*** to remove remaining cells. Plasma supernatant was transferred into a 1.5‐mL tube and stored at −80 °C until use.

### 2.4 DNA purification

Circulating free DNA (cfDNA) was performed with the QIAamp Circulating Nucleic Acid Kit (QIAGEN, Venlo, Netherlands) according to the manufacturer's instructions. DNA quality and concentration were measured with a NanoDrop 1000 Spectrophotometer (Thermo Scientific, Waltham, MA, USA).

### 2.5 Mutation detection by BEAMing technology in ctDNA

*RAS* status was determined in plasma using BEAMing (Sysmex Corporation, Kobe, Japan). The commercially available, and previously validated (Grasselli *et al.*, 2017), CE‐IVD BEAMing *RAS* plasma panel of mutations was evaluated (Table S2). Plasma was processed as previously described (Grasselli *et al.*, 2017). Samples were considered mutant according to a mutation rate threshold (0.02–0.04%) based on the CE‐IVD BEAMing *RAS* panel assay, as per the manufacturer's algorithm.

### 2.6 Statistics

Statistical analyses were performed using r 3.4.1, r studio (v. 1.0.153, https://www.r-project.org/), and the cran r survival package. Data are summarized by frequency for categorical variables and by median and range for continuous variables. PFS was defined as the time from treatment start to disease progression or death. OS was defined as the time from mCRC diagnosis to death from any cause or the last follow‐up visit. Response rate was assessed according to recist 1.1 (https://recist.eortc.org/).

Mutant allele fractions were calculated as the number of mutant beads divided by the total number of beads analyzed, and all samples were analyzed blinded to the study endpoints. Pearson's correlation coefficients between MAF levels and selected clinical variables were determined. Clinical variables analyzed included treatment line, primary site, number and location of metastatic sites, best response, carcinoembryonic antigen (CEA) and CA 19‐9 levels, number of metastatic hepatic lesions, and hepatic lesion volume (sum of the largest diameter of all hepatic lesions [maximum 10], according to recist v1.1). Significance was determined with nonparametric Kruskal–Wallis tests; *P*‐values < 0.05 were considered significant.

Hazard ratios (HR) and 95% confidence intervals (CI) were calculated. Survival curves were estimated using the Kaplan–Meier method. Log‐rank tests, including univariate and multivariate Cox proportional hazards models, were performed for key endpoints.

### 2.7 MAF cutoff

The optimal MAF cutoff of 5.8% used in our cohort was calculated based on our dataset using the r function cutp in the survmisc package (Contal and O'Quigley, 1999; Mandrekar *et al.*, 2003). This function determines the optimal cut point for a continuous variable in a coxph or survfit model under the null hypothesis that the chosen cutoff does not predict survival.

## 3 Results

### 3.1 Correlation of MAF with clinical parameters

**Figure 1:**
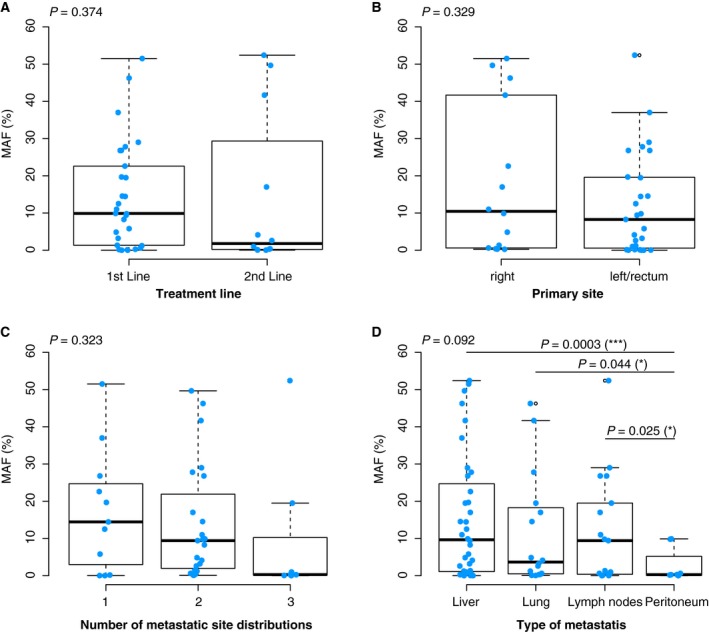
MAF distribution. Representation of MAF (%) distributions according to: (A) the two lines of treatment; (B) tumor laterality; (C) number of metastatic lesions; and (D) metastatic site. Box plots show the interquartile range (IQR) with median, 25th and 75th percentile, outliers, and *P*‐values. Continued lines (in graph D) indicate the comparison between two variables. Statistically significant *P*‐values are marked with a (*). Samples are represented by light blue dots.

A total of 41 samples from 37 patients were analyzed, 29 prior to first‐line and 12 prior to second‐line treatment. A wide range of plasma *RAS* MAFs was seen for both the first‐ and second‐line treatment groups. Median MAF was 9.9% in the first‐line group (range: 0.014–51.5%) and 1.8% (range: 0.03–52.4%) in the second‐line group (Fig. 1A), although not statistically significant. MAF distribution did not correlate with tumor right/left sidedness or the number of metastatic sites (Fig. 1B,C) but, in contrast, varied significantly according to the site of metastasis. MAF median values were calculated according to metastatic spread involving the liver, lung, lymph nodes, or peritoneum (most patients had more than one metastatic site). Median MAF was significantly lower in patients with metastases in the peritoneum compared to those with metastases in the liver (*P* = 0.0003), lung (*P* = 0.044), or lymph nodes (*P* = 0.025; Fig. 1D), although this observation is limited by sample size.

A multivariate Cox analysis showed that MAFs did not significantly correlate with CA19‐9 or CEA levels, though a slight tendency to higher levels of CA19‐9 was observed in samples with higher MAFs. CEA levels were overall higher than reference values; however, no association was observed with MAFs (Fig. S2, Table S3). Similarly, MAFs did not correlate with the number of metastatic hepatic lesions or hepatic volume (Fig. S2).

### 3.2 Correlation of MAF with clinical outcomes

Overall, higher MAF values correlated with shorter PFS (cor = −0.476; *P* = 0.009) and OS (cor = −0.506; *P* = 0.005; Fig. S3E). We decided to set a cutoff MAF value that split patients with better vs worse prognosis in our cohort. This was done with the Cutpoint function (*cutp*) for a continuous variable in a Coxph or Survfit model.

**Figure 2:**
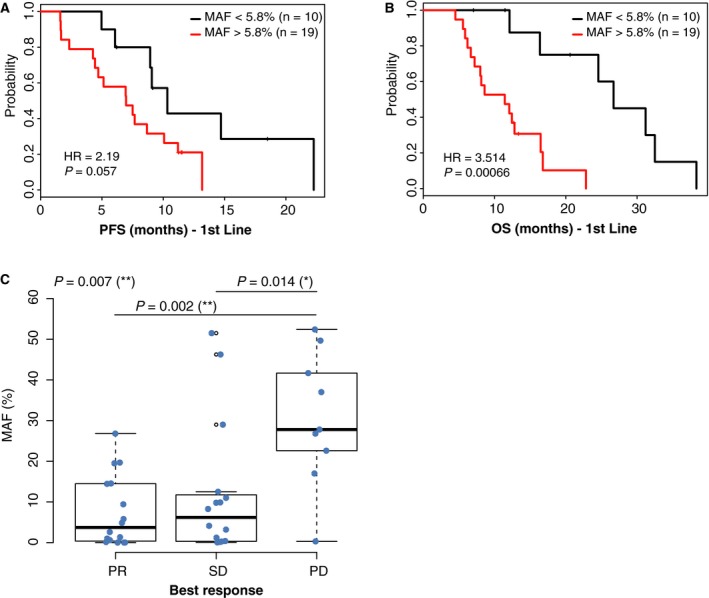
PFS and OS analyses in first‐line treatment. Survival curves are shown for samples with MAF < 5.8% (black line) and MAF> 5.8% (red line) in terms of PFS (A) and OS (B) in the 1st line. HR and *P‐*values are shown. (C) MAF distribution according to best response to treatment.

In the first‐line setting, using an optimized MAF cutoff of 5.8%, PFS was not significantly better in samples with MAF < 5.8% (Fig. 2A); however, a trend toward lower PFS was observed in samples with a higher MAF. The difference in median PFS between patients with *RAS*‐mutant samples with MAF < 5.8% (*N* = 10) and those with MAF ≥ 5.8% was 10.7 months vs 7.0 months with an HR of 2.2 (95% CI: 0.94–7.20; *P* = 0.06).

Using the same optimized cutoff of 5.8%, samples with MAF < 5.8% showed significantly better OS (Fig. 2B). The difference in median OS between patients with *RAS*‐mutant samples having MAF < 5.8% (*N* = 10) and those with MAF ≥ 5.8% was 26.7 months vs 11.4 months (HR: 3.5; 95% CI: 2.08–43.1; *P* = 0.0006). *RAS* MAF was still an independent variable for OS with a 1% cutoff, but at 10% cutoff, HR was no longer significant (Fig. S3A–D).

In the second‐line setting, the analyses show clearly that patients with MAF < 5.8% have both longer PFS and OS, with an HR of 6.6 and *P* = 0.00018 for both variables, compared to those with MAF > 5.8% (Fig. S4A,B). *RAS* MAF remained an independent variable with cutoffs of 1% or 10% (Fig. S4C,F).

Mutant allele fractions were significantly higher in patients whose outcome was progression disease (PD), compared to those with partial response (PR; Fisher's test *P* = 0.002) or stable disease (SD; Fisher's test *P* = 0.014; Fig. 2C). One‐way ANOVA test draws identical conclusions (*P* = 0.007).

Univariate analyses in the first‐line cohort including different clinical factors such as tumor location, number of metastatic sites, gender, and CEA levels showed plasma MAF was the only statistically significant prognostic factor for OS. Multivariate Cox analysis considering the previous biomarkers showed that plasma RAS MAF was the strongest prognostic factor for both PFS (HR: 3.74; 95% CI 1.01–13.92; *P* = 0.049) and OS (HR: 2.73; 95% CI 2.35–182.53; *P* = 0.006; Table 2).

**Table 2:** Multivariate analysis for first‐line PFS and OS.

Risk factor	PFS			OS		
	HR	95% CI	*P‐*value	HR	95% CI	*P‐*value
Gender	2.37	0.77–7.38	0.135	1.20	0.33–4.38	0.778
Laterality	0.54	0.17–1.74	0.303	0.32	0.08–1.19	0.088
CEA	0.99	0.99–1.00	0.656	0.99	0.99–1.00	0.711
No. of hepatic lesions	1.04	0.32–3.37	0.947	1.05	0.30–3.70	0.935
Plasma MAF	3.74	1.01–13.92	0.049	2.73	2.35–182.53	0.006

**Figure 3:**
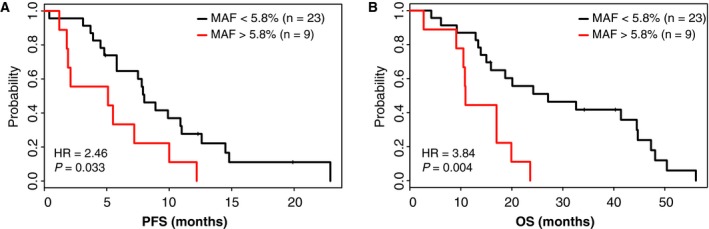
PFS and OS analyses in the validation cohort (CAPRI‐GOIM trial). Survival curves are shown for samples with MAF < 5.8% (black line) and MAF > 5.8% (red line) in terms of PFS (A) and OS (B). HR and *P‐*values are shown.

Consistent with our results, in an independent cohort at first‐line treatment from the CAPRI‐GOIM trial, samples with MAF < 5.8% showed significantly better OS (HR: 3.84; 95% CI 1.5–9.6; *P* = 0.004) and longer PFS (HR: 2.5; 95% CI: 1.07–5.62; *P* = 0.033; Fig. 3). Similar results were obtained when using a cutoff of 10% (Fig. S5).

## 4 Discussion

This is the first clinical study that aims at specifically assessing the prognostic potential of measuring *RAS* MAFs in cfDNA in a homogenous group of mCRC patients. Previously, we and others observed that patients with lower OS tend to have a plasma *RAS* MAF above 10% (El Messaoudi *et al.*, 2016; Grasselli *et al.*, 2017; Vidal *et al.*, 2017). The present study aimed to accurately define the impact of *RAS* MAF in a homogenous cohort of mutated mCRC patients, thereby excluding potential confounding factors, in the context of specific clinical parameters and survival outcomes. We correlated *RAS* MAF with several clinical characteristics including previously proposed prognostic biomarkers, such as laterality, CEA, CA19.9, hepatic tumor volume, number of metastatic sites, and previous lines of therapy, to gain a better understanding of the biological basis of plasma MAFs in mCRC patients. However, no linear correlations were found with any of these parameters—an outcome which warrants further research with expanded cohorts.

To date, there is plausible evidence that the primary tumor side might have prognostic value in mCRC (Arnold *et al.*, 2017; Petrelli *et al.*, 2017). Our multivariate analysis revealed that tumor sidedness was not a prognostic factor in our cohort. This apparent discordance with previous publications might be accounted for by a bias concerning the study populations; our cohort is relatively small and included only *RAS*‐mutated mCRC samples, whereas other studies were based on patients with *RAS* wild‐type mCRC (Arnold *et al.*, 2017) or did not take *RAS* mutational status into account (Petrelli *et al.*, 2017).

While MAF distribution was independent of hepatic tumor volume and the number of metastatic hepatic lesions, the presence of metastases in the liver, lung, or lymph nodes was significantly associated with higher MAFs. Although there is currently no clear explanation for this phenomenon, our results are in line with previous studies reporting that the site of metastatic spread rather than the number of lesions has prognostic value in mCRC (Riihimäki *et al.*, 2016; Vidal *et al.*, 2017; Yaeger *et al.*, 2015).

Evaluation of best response to treatment showed that patients with higher MAFs had PD or SD rather than PR. Our study also indicates that patients with higher RAS MAFs do present with more resistant tumors to conventional therapies. New or experimental approaches should be considered for them.

In our multivariate statistical model, *RAS* MAF did not correlate with either CEA or CA19‐9, unlike recent observation that elevated CA19‐9 levels represented a strong prognostic marker (Rahbari *et al.*, 2017). However, we did observe that patients with higher MAFs also tended to have higher levels of both CA19‐9 and CEA, though not reaching statistical significance.

The most striking finding of our analysis is that the estimation of *RAS* MAF in liquid biopsies correlates with predicting life expectancy in this mCRC population. Our data provide evidence that baseline patients with higher *RAS* MAFs in cfDNA tend to progress after a shorter time and have significantly shorter OS. An independent cohort from the CAPRI‐GOIM trial was analyzed, and plasma MAF at baseline resulted statistically significant for prognosis in both OS and PFS. An improved prognostic value in PFS was observed in this first‐line setting. A MAF cutoff was selected, using the *cutp* algorithm, based on the ability to better segregate outcomes in terms of PFS and OS (5.8% MAF). Additional cutoffs of 1% and 10% MAF used in previous publications (El Messaoudi *et al.*, 2016; Siravegna *et al.*, 2017; Vidal *et al.*, 2017) were also evaluated, being 5.8% the one that overall provided better prognostic value in our patient cohorts. The sample size is indeed relatively small and larger prospective studies to confirm our analyses and further evaluate clinical parameters will be valuable.

## 5 Conclusion

Our data strongly support that *RAS* MAFs have independent prognostic value for CRC survival and that, along with tumor and patient characteristics, could provide a useful noninvasive decision‐making tool in the first‐line setting. After demonstrating the feasibility for implementing liquid biopsies in routine care (Grasselli *et al.*, 2017), we propose *RAS* MAFs as a novel independent prognostic biomarker for mCRC.

## Conflict of interest

JT has served in a consulting or advisory role for Amgen, Boehringer Ingelheim, Celgene, Chugai, ImClone, Lilly, Merck, S.L., Madrid, Merck Serono, Millennium Pharmaceuticals, Inc., Novartis, Roche, Sanofi, and Taiho. RS has served in a consulting or advisory role for Amgen, Merck, S.L., Madrid, and Roche Dx and obtained research funding from Roche Dx. AV has served in a consulting or advisory role for Merck, S.L., Madrid, Merck Serono, and Sysmex. All remaining authors have declared no conflicts of interest.

## Author contributions

AV and EE conceived of the presented idea. AV, EE, TT, and FC developed the study design. ES, EM, AN, JG, GC, RE, GM, CS, TM, GA, JC, and NM contributed to sample acquisition. GC and JM performed the experiments. FM, AG, and AV performed the quality control of data and algorithms. FM, EE, AV, and RS contributed to data analyses and the interpretation of the results. FM, EM, and NN performed the statistical analysis. FM prepared the figures. CC wrote and edited the manuscript with support from EE and AV. All authors provided critical feedback and helped shape the research, analysis, and manuscript.

## Supporting information


**Fig. S1**. Sample selection. Flowchart of selection steps for the analysis population.

**Fig. S2**. Correlation analysis. Dot plots depicting the correlation between MAF (%) and the following parameters: CA19.9, CEA, hepatic volume and number of metastatic sites. Pearson correlation coefficient (cor) and *P*‐values are reported.

**Fig. S3**. PFS and OS analyses at different MAF cut‐offs in first‐line treatment. Survival curves are shown for samples with MAF < 1% (black line) and MAF > 1% (red line) in terms of PFS (A) and OS (B), as well as for samples with MAF < 10% (black line) and MAF > 10% (red line) in terms of PFS (C) and OS (D). HR and *P‐*values are shown. Correlation between PFS/OS and MAF is reported (E). Pearson correlation coefficient (cor) and the *P*‐value are reported.

**Fig. S4**. PFS and OS analyses in second‐line treatment. Survival curves are shown for samples with: (a) MAF < 5.8% (black line) and MAF > 5.8% (red line) in terms of PFS (A) and OS (B); (b) MAF < 1% (black line) and MAF > 1% (red line) in terms of PFS (C) and OS (D); (c) MAF < 10% (black line) and MAF > 10% (red line) in terms of PFS (E) and OS (F). HR and *P‐*values are shown.

**Fig. S5**. PFS and OS analyses at different MAF cut‐offs in the validation cohort (CAPRI‐GOIM trial). Survival curves are shown for samples with MAF < 1% (black line) and MAF > 1% (red line) in terms of PFS (A) and OS (B), as well as for samples with MAF < 10% (black line) and MAF > 10% (red line) in terms of PFS (C) and OS (D). HR and *P‐*values are shown.

**Table S1**. Description of patients used in the study.

**Table S2**. *RAS* panel of mutations for BEAMing analysis.

**Table S3**. MAF tendency according to CA 19‐9 and CEA levels.
