# Supplementary material for: Impact of circulating tumor DNA mutant allele fraction on prognosis in RAS‐mutant metastatic colorectal cancer
Source: Mol Oncol. 2019 Jul 31;13(9):1827–35. doi: 10.1002/1878-0261.12547 (PMC6717744; doi:10.1002/1878-0261.12547)
Supplement: Supplementary file 1 — Fig. S1. Sample selection. Flowchart of selection steps for the analysis population. [file MOL2-13-1827-s001.pdf]

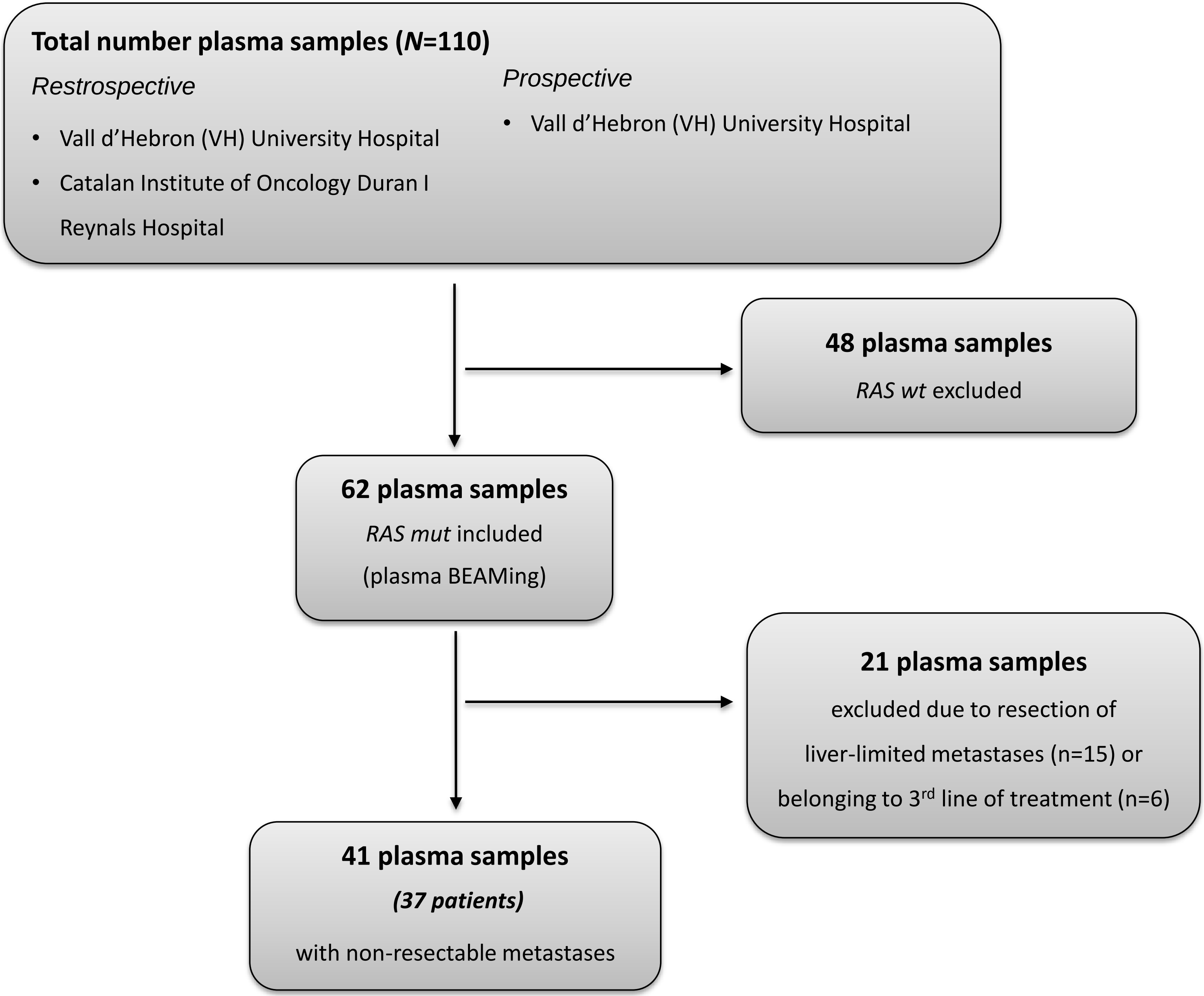

Total number plasma samples (N=110)
Restrospective
Vall d'Hebron (VH) University Hospital
Catalan Institute of Oncology Duran I Reynals Hospital
Prospective
Vall d'Hebron (VH) University Hospital
48 plasma samples
RAS wt excluded
62 plasma samples
RAS mut included
(plasma BEAMing)
21 plasma samples
excluded due to resection of
liver-limited metastases (n=15) or
belonging to 3rd line of treatment (n=6)
41 plasma samples
(37 patients)
with non-resectable metastases
