# Supplementary figures and images for: Impact of circulating tumor DNA mutant allele fraction on prognosis in RAS‐mutant metastatic colorectal cancer
Source: Mol Oncol. 2019 Jul 31;13(9):1827–35. doi: 10.1002/1878-0261.12547 (PMC6717744; doi:10.1002/1878-0261.12547)

**FIG. S2**

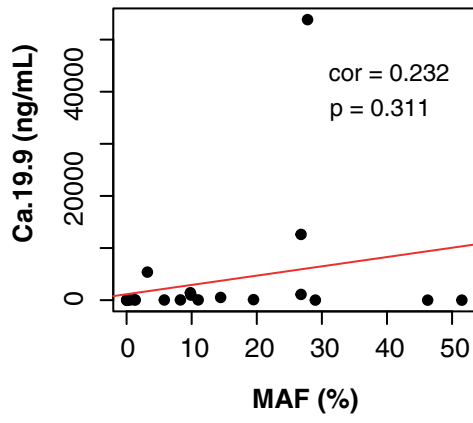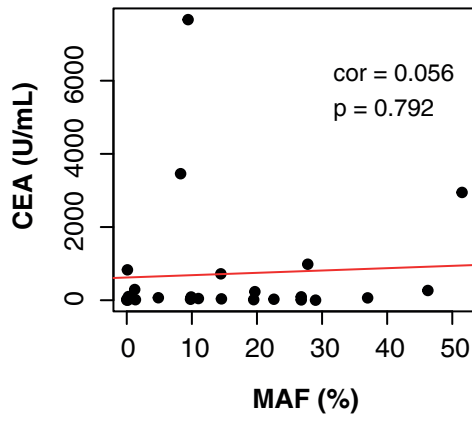

**serum  
biomarkers**

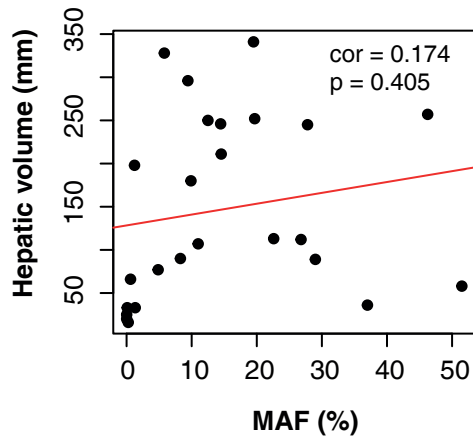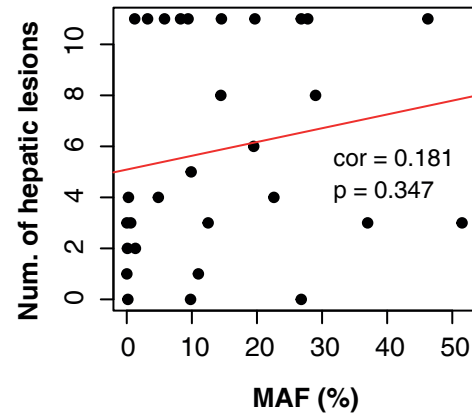

**tumor  
burden**

Supplement: Supplementary file 2 — Fig. S2. Correlation analysis. Dot plots depicting the correlation between MAF (%) and the following parameters: CA19.9, CEA, hepatic volume and number of metastatic sites. Pearson correlation coefficient (cor) and P‐values are reported. [file MOL2-13-1827-s002.pdf]

FIG.S3

A

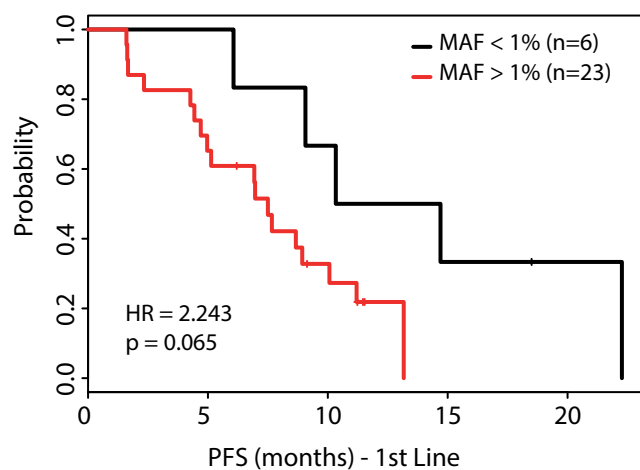

B

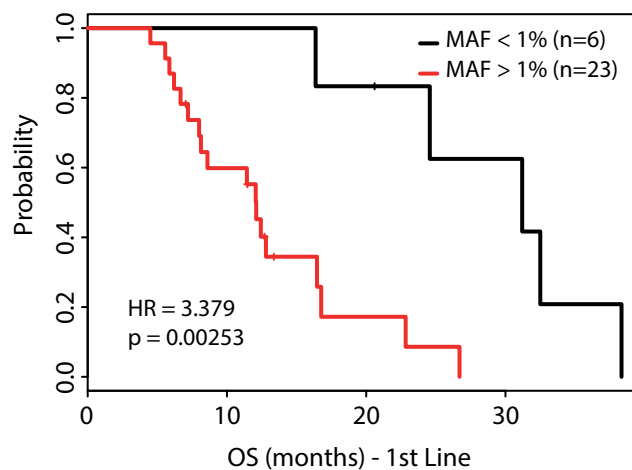

C

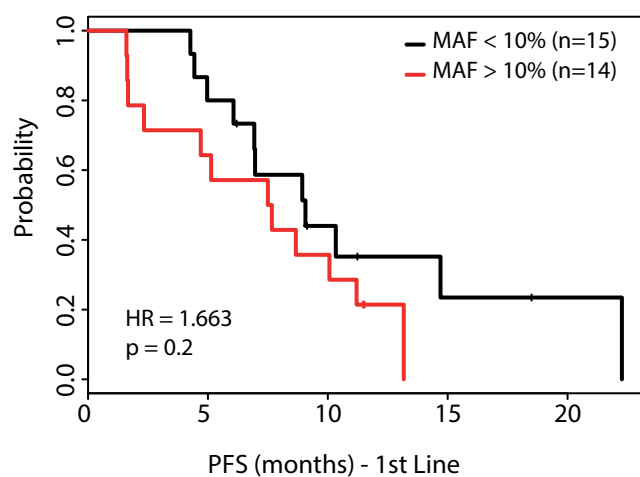

D

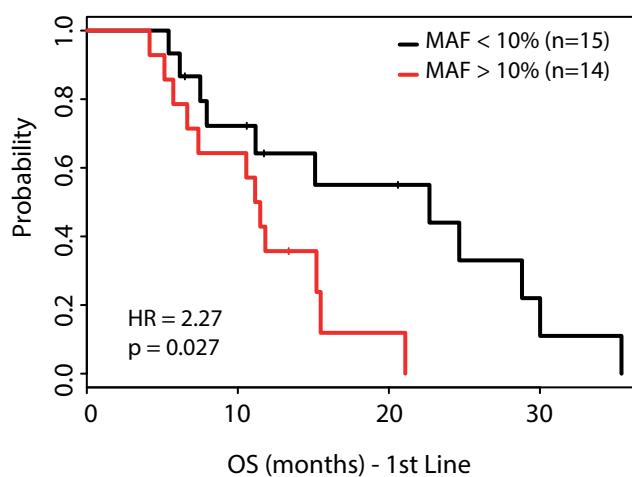

E

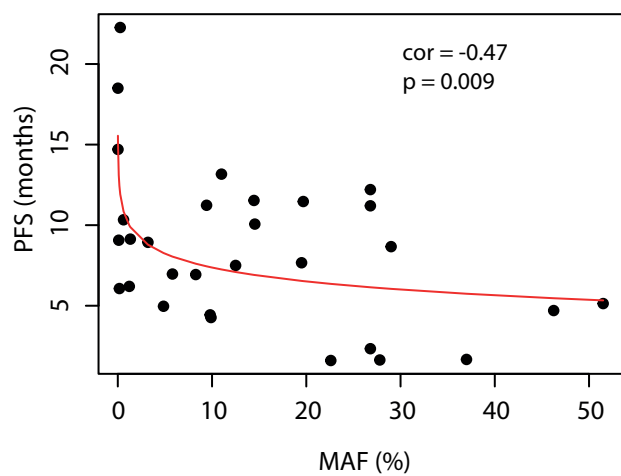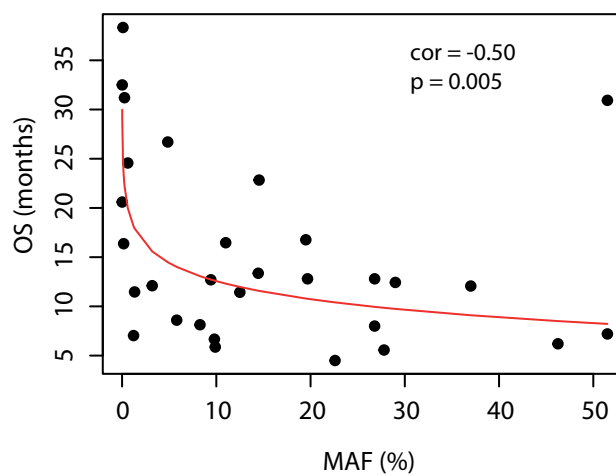

Supplement: Supplementary file 3 — Fig. S3. PFS and OS analyses at different MAF cut‐offs in first‐line treatment. Survival curves are shown for samples with MAF < 1% (black line) and MAF > 1% (red line) in terms of PFS (A) and OS (B), as well as for samples with MAF < 10% (black line) and MAF > 10% (red line) in terms of PFS (C) and OS (D). HR and P‐values are shown. Correlation between PFS/OS and MAF is reported (E). Pearson correlation coefficient (cor) and the P‐value are reported. [file MOL2-13-1827-s003.pdf]

FIG. S4

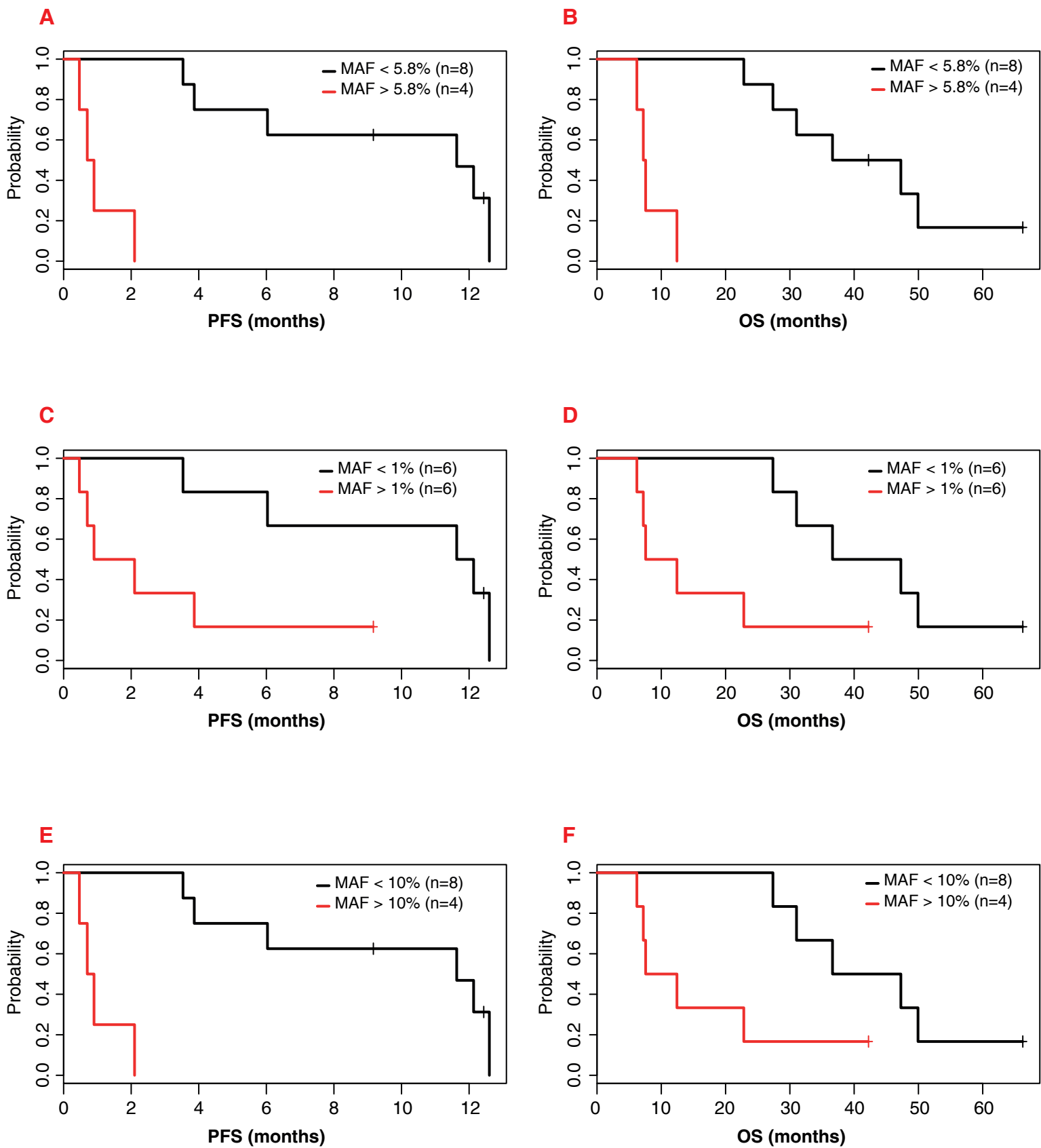

Supplement: Supplementary file 4 — Fig. S4. PFS and OS analyses in second‐line treatment. Survival curves are shown for samples with: (a) MAF < 5.8% (black line) and MAF > 5.8% (red line) in terms of PFS (A) and OS (B); (b) MAF < 1% (black line) and MAF > 1% (red line) in terms of PFS (C) and OS (D); (c) MAF < 10% (black line) and MAF > 10% (red line) in terms of PFS (E) and OS (F). HR and P‐values are shown. [file MOL2-13-1827-s004.pdf]

FIG. S5

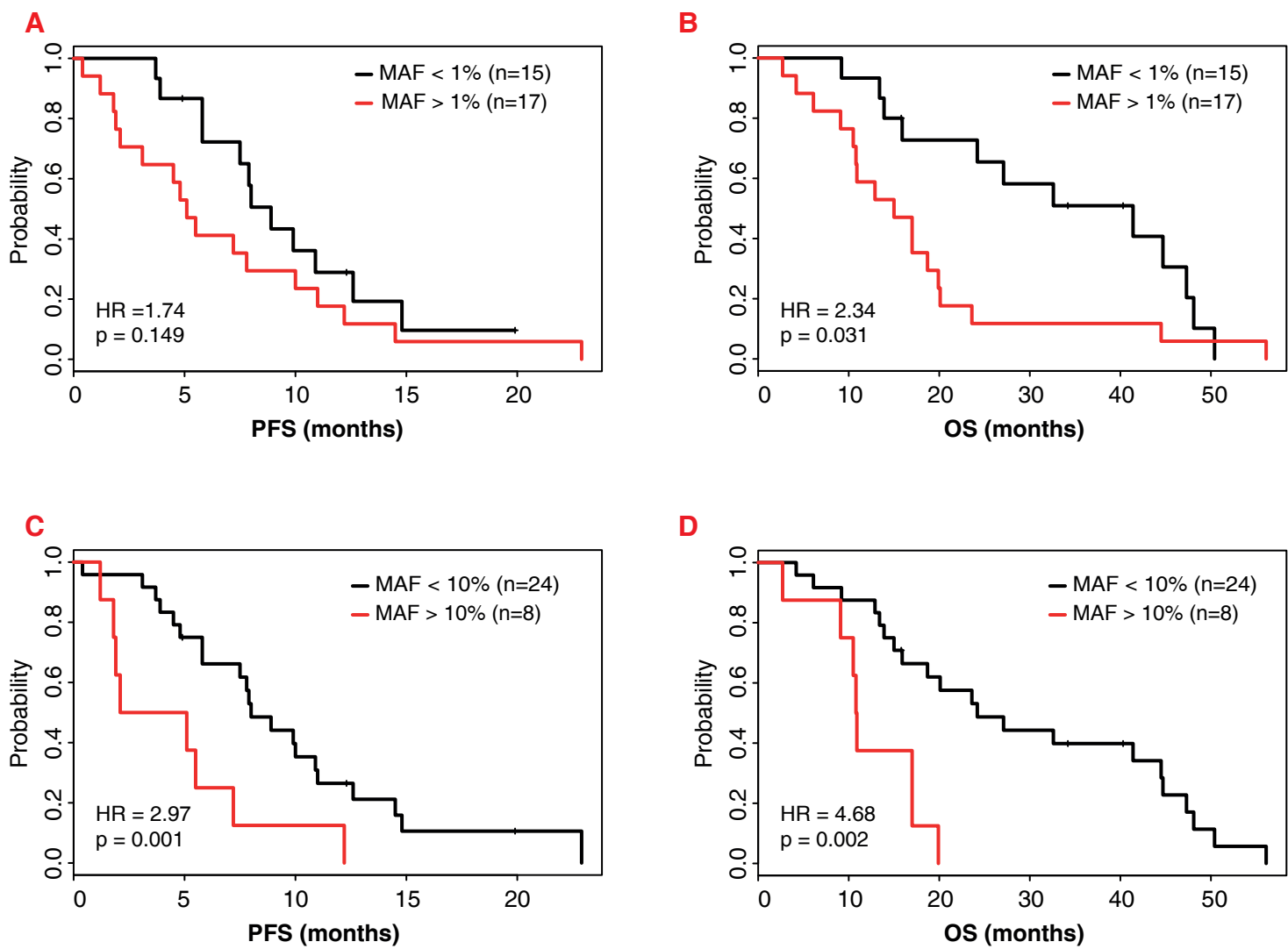

Supplement: Supplementary file 5 — Fig. S5. PFS and OS analyses at different MAF cut‐offs in the validation cohort (CAPRI‐GOIM trial). Survival curves are shown for samples with MAF < 1% (black line) and MAF > 1% (red line) in terms of PFS (A) and OS (B), as well as for samples with MAF < 10% (black line) and MAF > 10% (red line) in terms of PFS (C) and OS (D). HR and P‐values are shown. [file MOL2-13-1827-s005.pdf]
