# Supplementary material for: Impact of circulating tumor DNA mutant allele fraction on prognosis in RAS‐mutant metastatic colorectal cancer
Source: Mol Oncol. 2019 Jul 31;13(9):1827–35. doi: 10.1002/1878-0261.12547 (PMC6717744; doi:10.1002/1878-0261.12547)
Supplement: Supplementary file 6 — Table S1. Description of patients used in the study. [file MOL2-13-1827-s006.docx]

**VHIO cohort: Patients in first line treatment**

| **Patient** | **Sex** | **Tumor Grading** | **Metastatic status at diagnosis** | **Tumor location** | **Response** | **PFS months** | **OS months** | **Tissue NGS results** | **Plasma Beaming Results (VarFreq)** | **Prior surgery** | **Site of metastasis** |
| --- | --- | --- | --- | --- | --- | --- | --- | --- | --- | --- | --- |
| s1 | M | UNK | No | right | PR | 10,3 | 24,6 | KRAS A146T | KRAS A146T (0.61%) | Yes | Liver, Lung, Peritoneal, Lymph node |
| s2 | F | UNK | Yes | left | SD | 9,1 | 38,3 | KRAS G13D | KRAS G13D (0.1%) | Yes | Liver, lung |
| s3 | F | UNK | Yes | left | PR | 7 | 8,6 | KRAS G12D | KRAS G12D (5.8%) | No | Liver |
| s4 | M | UNK | Yes | rectum | SD | 8,9 | 12,1 | KRAS G12D | KRAS G12D (3.2%) | No | Liver, lung |
| s5 | M | UNK | Yes | rectum | SD | 7,5 | 11,4 | KRAS G12D | KRAS G12D (12.5%) | No | Liver |
| s6 | M | G2 | Yes | rigth | SD | 22,3 | 31,2 | KRAS G12D | KRAS G12D (0.26%) | Yes | Liver, Peritoneal, Splenic |
| s7 | M | G2 | Yes | left | PD | 1,7 | 12,1 | KRAS G12V | KRAS G12V (37%) | Yes | Liver |
| s8 | F | G2 | Yes | left | PD | 1,6 | 5,6 | KRAS G12V | KRAS G12V (27.8%) | No | Liver, lung |
| s9 | M | G2 | Yes | left | PR | 14,7 | 32,5 | KRAS G12V | KRAS G12V (0.02%) | Yes | Liver |
| s10 | M | G2 | No | rigth | SD | 13,2 | 16,5 | NRAS Q61R | NRAS Q61R (11%) | Yes | Liver, lymph node |
| s11 | M | G3 | No | rectum | SD | 6,9 | 8,1 | NRAS Q61R | NRAS Q61R (8.27%) | Yes | Liver, Bone |
| s12 | M | G1 | Yes | rectum | SD | 6,1 | 16,4 | KRAS G12D | KRAS G12D (0.17%) | Yes | Peritoneal |
| s13 | M | G3 | Yes | right | SD | 4,3 | 5,9 | NRAS G12D | NRAS G12D (9.89%) | Yes | Liver, Peritoneal |
| s14 | F | G3 | Yes | rigth | PD | 1,6 | 4,5 | KRAS G12V | KRAS G12V (22.6%) | Yes | Liver |
| s15 | M | G1 | Yes | rigth | PR | 5 | 26,7 | KRAS G13D | KRAS G13D (4.85%) | Yes | Liver, lung |
| s16 | F | G3 | Yes | left | PD | 2,3 | 8 | KRAS G13D | KRAS 13 (26.8%) | Yes | Liver, lymph node |
| s17 | M | G2 | Yes | rectum | SD | 6,2 | 7 | WT | KRAS 12 (1.22%) | No | Liver, lung |
| s18 | F | G3 | Yes | rectum | PR | 7,7 | 16,8 | KRAS G12C | KRAS 12 (19.5%) | No | Liver, lung, lymph node |
| s19 | M | UNK | Yes | left | PR | 11,2 | 12,7 | NRAS Q61L | KRAS 61 (9.42%) | No | Liver, lymph node |
| s20 | M | UNK | Yes | left | PR | 11,5 | 12,8 | KRAS G12D | KRAS 12 (19.68%) | No | Liver |
| s21 | F | UNK | Yes | left | SD | 4,4 | 6,7 | KRAS G13D | KRAS 13 (9.8%) | No | Peritonel, Lymph node |
| s22 | M | UNK | Yes | left | PR | 11,5 | 13,4 | NRAS Q61L | NRAS 61 (14.45%) | No | Liver |
| s23 | F | G1 | Yes | right | PR | 9,1 | 11,5 | KRAS G13D | KRAS 13 (1.33%) | Yes | Liver, Lymph node, |
| s24 | F | G3 | Yes | left | PR | 11,2 | 12,8 | KRAS G12W | KRAS 12 (26.8%) | Yes | Lymph node |
| s25 | M | G3 | Yes | right | SD | 4,7 | 6,2 | KRAS G13D | KRAS G13 (46.25%) | No | Liver, Lung |
| s26 | M | G3 | Yes | left | PR | 18,5 | 20,6 | KRAS G12 | KRAS G12 (0.014%) | Yes | Liver, Peritoneal, Lymph node |
| s27 | M | G1 | Yes | rectum | SD | 8,7 | 12,4 | KRAS G12 | KRAS G12 (29%) | No | Liver, Lymph node |
| s28 | M | G1 | Yes | right | SD | 5,1 | 7,2 | KRAS G12 | KRAS G12 (51.5%) | No | Liver |
| s29 | F | G1 | Yes | rectum | PR | 10,1 | 22,8 | KRAS A146 | KRAS A146 (14.54%) | No | Liver, Lung |

**VHIO cohort: Patients in second line treatment**

| **Patient** | **Sex** | **Tumor Grading** | **Metastatic status at diagnosis** | **Tumor location** | **Response** | **PFS2 months** | **OS months** | **Tissue NGS results** | **Plasma Beaming Results (VarFreq)** | **Prior surgery** | **Site of metastasis** |
| --- | --- | --- | --- | --- | --- | --- | --- | --- | --- | --- | --- |
| s30 | F | UNK | No | rectum | PR | 12,4 | 66,2 | NRAS Q61K | NRAS Q61K (0.03%) | Yes | Lymph node |
| s31 | F | G2 | Yes | rectum | PR | 11,6 | 49,9 | KRAS G12C | KRAS G12C (0.138%) | Yes | Liver |
| s32 | M | UNK | Yes | right | PD | 3,5 | 47,3 | KRAS G12C | KRAS G12C (0.3%) | Yes | Liver, Lung, Peritoneal, Lymph node |
| s33 | F | UNK | Yes | rectum | PR | 12,1 | 27,4 | KRAS G13D | KRAS G13D (0.99%) | No | Liver, Lymph node, Adrenal |
| s34 | F | G3 | Yes | rectum | PR | 9,2 | 42,2 | KRAS G12C | KRAS G12C (2.6%) | Yes | Liver, Lung |
| s29 | F | UNK | Yes | rectum | SD | 3,9 | 22,8 | KRAS A146 | KRAS A146 (4.129%) | No | Liver, Lung |
| s35 | M | UNK | Yes | rigth | PD | 0,5 | 7,6 | KRAS G13D | KRAS G13D (17%) | No | Liver, lung, lymph node |
| s25 | M | UNK | Yes | right | PD | 0,7 | 6,2 | KRAS G13D | KRAS G13 (41.67%) | No | Liver, Lung |
| s28 | M | G1 | Yes | right | PD | 0,9 | 7,2 | KRAS G12 | KRAS G12 (49.66%) | No | Liver |
| s27 | M | G1 | Yes | rectum | PD | 2,1 | 12,4 | KRAS G12 | KRAS G12 (52.4%) | No | Liver, Lymph node |
| s36 | M | UNK | No | rectum | SD | 12,6 | 31 | KRAS G12A/ NRAS Q61 | KRAS G12A (0.058%) | Yes | Liver, Lung, Peritoneal, Lymph node |
| s37 | M | UNK | Yes | right | SD | 6 | 36,6 | KRAS G12D/NRAS G12V | KRAS G12D/NRAS G12V (0.2%/0.39% ) | Yes | Lung, Lymph node |

**Patients enrolled by the CAPRI-GOIM trial and plasma mutated**

| **Patient** | **Sex** | **Tumor Grading** | **Metastatic status at diagnosis** | **Tumor location** | **Response** | **PFS months** | **OS months** | **Tissue NGS results** | **Plasma Beaming Results (% mut Beads)** | **Prior surgery** | **Site of metastasis** |
| --- | --- | --- | --- | --- | --- | --- | --- | --- | --- | --- | --- |
| CAPRI_001 | F | G2 | Yes | Left | SD | 12,3 | 34.2* | WT | KRAS: codon 12 (0.026%) | Yes | Liver |
| CAPRI_002 | F | G3 | Yes | Right | PR | 8 | 32,6 | WT | KRAS: codon 12 (0.056%) | Yes | Liver, limph node |
| CAPRI_003 | M | G2 | Yes | Left | SD | 12,6 | 50,4 | WT | KRAS: codon 12 (0.078%) | Yes | Liver, spleen |
| CAPRI_004 | F | G2 | No | Right | PR | 7,5 | 41,4 | KRAS: p.G12V (c.35G>T) (6.1%) | KRAS:codon 12 (0.127%) | Yes | Lymph node |
| CAPRI_005 | F | G2 | Yes | Left | PR | 10,9 | 27,1 | KRAS: p.G12V (c.35G>T) (28%) | KRAS: codon 12 (0.132%) | Yes | Liver |
| CAPRI_006 | M | G2 | Yes | Left | PR | 5,8 | 47,3 | WT | KRAS: codon 12 (0.133%) | Yes | Liver |
| CAPRI_007 | F | G2 | No | Rectum | SD | 5,8 | 44,7 | KRAS: p.G12S (c.34G>A) (20.1%) | KRAS: codon 12 (0.198%) | Yes | Liver, limph node |
| CAPRI_008 | F | Gx | Yes | Rectum | SD | 3,9 | 9,2 | WT | KRAS: codon 61 (0.242%) | Yes | Liver |
| CAPRI_009 | M | G2 | Yes | Left | PR | 19,9 | 40.3* | WT | KRAS: codon 12 (0.607%) | Yes | Liver |
| CAPRI_010 | M | G2 | Yes | Left | PR | 3,7 | 13,4 | WT | KRAS: codon 12 (0.857%) | Yes | Liver |
| CAPRI_011 | M | G2 | No | Left | PR | 4,9 | 15.8* | KRAS: p.A146T (c.436G>A) (38%) | KRAS: codon 146 (0.859%) | Yes | Liver |
| CAPRI_012 | M | G2 | Yes | Right | SD | 9,9 | 15,9 | KRAS: p.G12V (c.35G>T) (29%) | KRAS: codon 12 (0.977%) | Yes | Liver, limph node |
| CAPRI_013 | M | G1 | Yes | Left | PR | 7,9 | 24,2 | NRAS: p.Q61L (c.182A>T) (37%) | NRAS: codon 61 (0.985%) | Yes | Liver |
| CAPRI_014 | F | G2 | Yes | Left | SD | 7,8 | 15 | NRAS: p.Q61L (c.182A>T) (41%) | NRAS: codon 61 (1.156%) | Yes | Liver |
| CAPRI_015 | M | G2 | Yes | Rectum | SD | 3,1 | 4,2 | KRAS: p.G12V (c.35G>T) (40%) | KRAS: codon 12 (1.207%) | Yes | Limph node |
| CAPRI_016 | M | G3 | Yes | Rectum | SD | 4,8 | 12,9 | KRAS: p.G13D (c.38G>A) (31%) | KRAS: codon 13 (1.938%) | Yes | Liver |
| CAPRI_017 | M | G3 | Yes | Rectum | PR | 11 | 20,1 | KRAS: p.A146T (c.436G>A) (37%) | KRAS: codon 146 (2.27%) | Yes | Liver, lung |
| CAPRI_018 | F | G2 | No | Left | PR | 4,5 | 18,7 | WT | KRAS: codon 12 (2.372%) | Yes | Lymph node, adrenal gland |
| CAPRI_019 | M | G2 | Yes | Left | PR | 14,5 | 44,5 | MUT | NRAS: codon 12 (3.45%) | Yes | Liver, Lung |
| CAPRI_020^b^ | F | G3 | Yes | Left | PR | 8,4 | 84.9* | KRAS: p.A146T (c.436G>A) (51%) | KRAS: codon 146 (8.924%) | Yes | Limph node |
| CAPRI_021 | F | UNK | Yes | Left | PD | 1,8 | 10,9 | MUT | KRAS: codon 12 (10.4%) | Yes | Liver, limph node |
| CAPRI_022 | M | G3 | Yes | Rectum | PR | 1,2 | 2,7 | KRAS: p.G12C (c.34G>T) (47%) | KRAS: codon 12 (10.852%) | Yes | Liver, limph node |
| CAPRI_023 | F | GX | Yes | Right | PD | 2,1 | 10,5 | KRAS: p.G12V (c.35G>T) (26%) | KRAS: codon 12 (12.65%) | Yes | Lung |
| CAPRI_024 | F | G2 | Yes | Right | CR | 7,2 | 19,9 | MUT | KRAS: codon 12 (13.5%) | Yes | Liver, limph node |
| CAPRI_025 | F | G3 | Yes | Rectum | SD | 5,1 | 9,1 | MUT | NRAS: codon 61 (14.6 %) | Yes | Liver, lung |
| CAPRI_026 | F | G2 | Yes | Rectum | SD | 5,5 | 10,8 | KRAS: p.G12R (c.34G>C) (19.4%) | KRAS: codon 12 (14.6%) | Yes | Liver, lung , limph node |
| CAPRI_027 | M | G3 | Yes | Rectum | SD | 1,9 | 17 | MUT | KRAS: codon 61 (16.448%) | Yes | Liver |
| CAPRI_028 | F | G1 | Yes | Left | PR | 8,9 | 13,9 | KRAS: p.A146T (c.436G>A) (9%) | KRAS: codon 146 (0.688%) | No | Liver, pelvis |
| CAPRI_029 | M | G2 | No | Right | PD | 0,4 | 6,1 | KRAS: p.G13D (c.38G>A) (74%) | KRAS: codon 13 (3.599%) | No | Liver |
| CAPRI_030 | M | Gx | Yes | Rectum | PR | 22,9 | 56,1 | WT | KRAS: codon 12 (5.619%) | No | Liver, lymph node |
| CAPRI_031 | M | G2 | Yes | Right | SD | 10 | 23,6 | MUT | NRAS codon 12 (5.982%) | No | Liver |
| CAPRI_032 | F | G1 | Yes | Left | SD | 12,2 | 17 | MUT | KRAS: codon 146 (27.4%) | No | Lung, pelvis |
| CAPRI_033 | F | G2 | Yes | Left | PR | 14,8 | 48,1 | WT | KRAS: codon 12 (0.488%) | UNK | Liver, lung, lymph node |

^a^) Before CAPRI enrollment and blood drawing.

^b^) This patient was excluded from the analyses due to the lack of clinical follow up.

^*^) censored cases 30 April 2017
