# Supplementary material for: Impact of circulating tumor DNA mutant allele fraction on prognosis in RAS‐mutant metastatic colorectal cancer
Source: Mol Oncol. 2019 Jul 31;13(9):1827–35. doi: 10.1002/1878-0261.12547 (PMC6717744; doi:10.1002/1878-0261.12547)
Supplement: Supplementary file 7 — Table S2. RAS panel of mutations for BEAMing analysis. [file MOL2-13-1827-s007.docx]

| **Supplementary Table S2: *RAS* panel of mutations for BEAMing analysis** | | |
| --- | --- | --- |
| **GEN** | **EXON** | **MUTATION** |
| *KRAS* | 2  2  3  3  4  4 | G12S/R/C/D/A/V  G13D  A59T  Q61L/H/R  K117N  A146T/V |
| *NRAS* | 2  2  3  3  4  4 | G12S/R/C/D/A/V  G13D/R/V  A59T  Q61K/L/R/H  K117N  A164T |
