# Supplementary material for: Impact of circulating tumor DNA mutant allele fraction on prognosis in RAS‐mutant metastatic colorectal cancer
Source: Mol Oncol. 2019 Jul 31;13(9):1827–35. doi: 10.1002/1878-0261.12547 (PMC6717744; doi:10.1002/1878-0261.12547)
Supplement: Supplementary file 8 — Table S3. MAF tendency according to CA 19‐9 and CEA levels. [file MOL2-13-1827-s008.docx]

| Supplementary Table S3. MAF tendency according to CA 19-9 and CEA levels. | | | |
| --- | --- | --- | --- |
| SampleID | **CA 19-9 (U/mL)** | **CEA (ng/mL)** | **MAF** |
| *s26* | 25.7 | 4.00 | 0.01% |
| *s9* | 8.5 | 25.10 | 0.02% |
| *s2* | 0.6 | 830.30 | 0.10% |
| *s12* | N/A | 4.60 | 0.17% |
| *s6* | 50.4 | 99.60 | 0.26% |
| *s1* | 48 | N/A | 0.61% |
| *s17* | 77.9 | 292.20 | 1.22% |
| *s23* | 35.2 | 14.80 | 1.33% |
| *s4* | 5385 | N/A | 3.20% |
| *s15* | N/A | 67.60 | 4.85% |
| *s3* | 34 | N/A | 5.80% |
| *s11* | 45.2 | 3458.00 | 8.27% |
| *s19* | N/A | 7669.00 | 9.42% |
| *s21* | 1425.8 | 26.90 | 9.80% |
| *s13* | 975.3 | 88.40 | 9.89% |
| *s10* | 49.6 | 46.30 | 11.00% |
| *s5* | N/A | N/A | 12.50% |
| *s22* | 522.9 | 721.40 | 14.45% |
| *s29* | N/A | 37.20 | 14.54% |
| *s18* | 99 | 15.00 | 19.50% |
| *s20* | N/A | 233.50 | 19.68% |
| *s14* | N/A | 26.80 | 22.60% |
| *s16* | 12615.4 | 92.00 | 26.80% |
| *s24* | 1104 | 9.10 | 26.80% |
| *s8* | 53832 | 984.20 | 27.80% |
| *s27* | 14.6 | 1.80 | 29.00% |
| *s7* | N/A | 64.10 | 37.00% |
| *s25* | 2 | 265.90 | 46.25% |
| *s28* | 0.6 | 2946.00 | 51.50% |

N/A, not available
